# A Review and Clarification of the Terms “holistic,” “configural,” and “relational” in the Face Perception Literature

**DOI:** 10.3389/fpsyg.2012.00559

**Published:** 2012-12-17

**Authors:** Daniel W. Piepers, Rachel A. Robbins

**Affiliations:** ^1^School of Social Sciences and Psychology, University of Western SydneySydney, NSW, Australia

**Keywords:** holistic, configural, relational, moving faces, composite task, part-whole task, inversion

## Abstract

It is widely agreed that the human face is processed differently from other objects. However there is a lack of consensus on what is meant by a wide array of terms used to describe this “special” face processing (e.g., holistic and configural) and the perceptually relevant information within a face (e.g., relational properties and configuration). This paper will review existing models of holistic/configural processing, discuss how they differ from one another conceptually, and review the wide variety of measures used to tap into these concepts. In general we favor a model where holistic processing of a face includes some or all of the interrelations between features and has separate coding for features. However, some aspects of the model remain unclear. We propose the use of moving faces as a way of clarifying what types of information are included in the holistic representation of a face.

In the field of visual perception, it is generally agreed that faces are processed differently to most other objects in that they are processed “holistically.” However there is a lack of consensus and clarity in the literature regarding what is meant by holistic processing and how it is different from the part-based processing most commonly attributed to the perception of non-face objects. Discussions of “whole” and “part” processing are in fact common across different areas of perception (i.e., visual, auditory, tactile), however it is not often made clear what constitutes a part and whole and how to differentiate them (Latimer and Stevens, 1997). This review article aims to address the lack of consensus and clarity in what is meant by parts and wholes in visual processing of faces and other objects. To do so we will first discuss the basis of the term “holistic” in Gestalt psychology and, through doing so, discuss how face perception may be qualitatively different from the perception of other objects. Secondly, we will discuss different ways in which configural and holistic processing have been conceptualized in the face perception literature and draw attention to alternate views of what is included in the “holistic representation” of a face (e.g., Maurer et al., 2002; Rossion, 2008; McKone and Yovel, 2009; Yovel, 2009). This will be followed by an overview of direct and indirect measures of holistic processing and a brief discussion about other aspects of face (e.g., attractiveness judgments; Abbas and Duchaine, 2008) and whole body perception (e.g., Robbins and Coltheart, 2012a,b) to which measures of holistic processing have been successfully applied as well as a brief overview of the developmental aspects of holistic processing (more detailed reviews can be found in McKone et al., 2012). We will finish by discussing how using moving faces in conjunction with common measures of holistic processing might help to clarify some of these issues and allow facial processing to be explored in a more naturalistic way.

## How Do We Perceive Objects and Faces?

It is generally agreed that most objects are processed on the basis of their individual parts or components (e.g., Biederman, 1987). A part-based perceptual model is useful as it allows: objects which cannot be seen in their entirety, from a single viewpoint, to be recognized based on their visible components; objects capable of engaging in non-rigid motion to be recognized easily regardless of part configuration (e.g., when rotated) and; objects to be perceived in a similar way to how we usually describe them (e.g., “that dog has pointy ears!”; Hoffman and Richards, 1984). However faces are thought to be processed in a qualitatively different way to most other objects (including objects of expertise; see Robbins and McKone, 2007; McKone and Robbins, 2011 for reviews). Evidence of this can be seen in behavioral studies where modified facial stimuli produce specific effects while other objects, that undergo the same manipulation, do not (e.g., Tanaka and Farah, 1993; Robbins and McKone, 2007). The difference has also been demonstrated in neuroimaging studies that have located specific face processing areas of the brain (see Kanwisher and Yovel, 2006 for review) and neuropsychological studies that have shown a double dissociation between specific impairments in the recognition of faces (i.e., prosopagnosia) and non-face objects (i.e., object agnosia; see Duchaine et al., 2006 for a review).

Face perception is different to the perception of other objects in that it is more “holistic.” Holism is the central premise of Gestalt theory which argues Gestalts are sensory wholes that are qualitatively different to the sum of their individual parts or components in that they “possess properties that cannot be derived from the properties of their constituent parts” (Wagemans et al., 2012b, p. 2). These properties are referred to as *emergent features*; an example of an emergent feature is the area of a square. A square possesses an area because its basic components (four lines of equal length) form an enclosed area that none of the lines can possess on their own. In the face perception literature terms such as configural, relational, and holistic are used to describe the emergent features of a face that only become apparent when two or more of its basic features (e.g., the eyes, nose, or mouth) are processed at the same time. What follows is a more comprehensive description of the wide array of terms used in the face perception literature.

## Models of Configural/Relational and Holistic Processing

Face perception differs from the perception of most other objects in that it relies heavily on emergent features (the interrelations between the more salient features of a face) as well as the features themselves. These emergent face features are often referred to as relational (e.g., Diamond and Carey, 1986) or configural information (e.g., Bartlett et al., 2003). These terms are used interchangeably here as we consider them to have fundamental underlying similarities and in the following sections we will attempt to show how these two terms have been used in similar ways. We will also describe how these terms relate to the concept of holistic processing.

Faces possess two types of relational/configural information: the *first-order relational properties* or first-order configuration refers to the basic configuration of the features within the face (e.g., eyes above nose, nose above mouth) while the *second-order relational properties* or second-order configuration refers to variations in the spacing between and positioning of the features (Diamond and Carey, 1986). The first-order configuration is thought to be important for detecting a face while the second-order configuration is important for discriminating between individual faces (e.g., Diamond and Carey, 1986; Tsao and Livingstone, 2008). It is the second-order configuration that is normally referred to when discussing relational/configural information. Sensitivity to face-like first-order configuration seems to be present from birth (e.g., Johnson et al., 1991).

**Figure 1:**
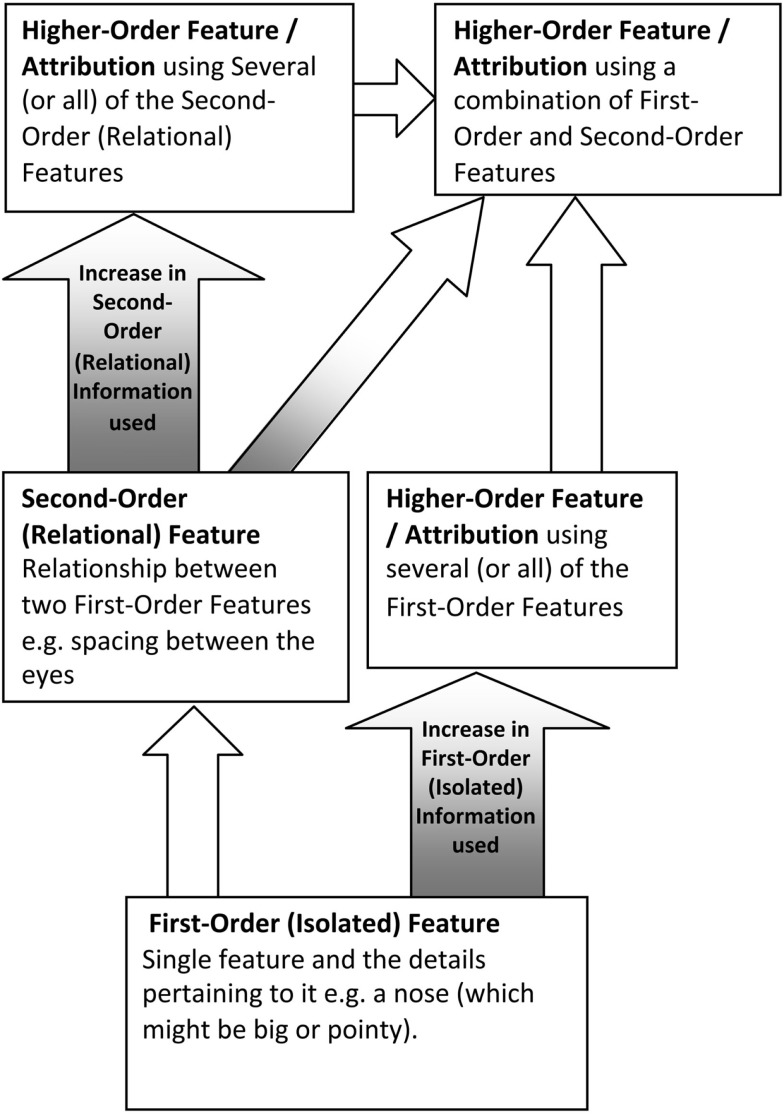
**An interpretation of a *relational/configural model* of face perception integrating the Diamond and Carey (1986) and Rhodes (1988) models**. The model starts with first-order (isolated) features (that are processed in a part-based manner), moves on to the emergence of second-order (relational) features (that are subject to configural processing), and finishes with increasingly complex higher-order features (attributions) which can involve a combination of first and/or second-order features.

A *relational/configural model* of face perception (e.g., Diamond and Carey, 1986; Rhodes, 1988; see Figure 1) is hierarchical in nature as it suggests that different types of judgments that can be made from faces (e.g., identity, expression, and attractiveness) can vary in the amount of second-order relational information needed to make them. According to Diamond and Carey (1986), information that can be used to discriminate between faces (i.e., identity) can be placed on a continuum ranging from isolated to relational features. Facial information that is relatively *isolated* (e.g., hair color) can be focused on without attending to information from other parts of the face. On the other hand, facial information that is acquired through processing two or more parts of a face simultaneously is said to be *relational* (as noted above, e.g., metric distance between the eyes). In a similar model proposed by Rhodes (1988), the cues used to discriminate individual faces are broken down into first-order, second-order, and higher-order features. *First-order features*, like isolated features, are those most salient features that can be processed independently of others (e.g., eyes, nose, mouth). *Second-order features*, like features at the relational end of Diamond and Carey’s (1986) continuum, are configural in nature and refer to individuating information acquired through processing two parts of a face simultaneously (e.g., spacing between the eyebrows and hairline, i.e., the forehead); while *higher-order features* (attributions) require a combination of several first-order and/or second-order features (e.g., age; Rhodes, 1988). The relational value of these *higher-order features* could be argued to vary depending on the number of second-order features included in them. Configural processing could best be described as the integration of all, or just some, of this second-order configural information within the face (Leder and Bruce, 2000; Bartlett et al., 2003). Individual pieces of configural (spacing) information may remain relatively variant or invariant while an individual face engages in different types of movement. For example the distance between the inner (or outer corners) of both eyes would remain fixed in cases of non-rigid (elastic) motion in the face but would change considerably with changes in facial viewpoint. The hierarchical nature of the *relational/configural model* suggests that configural processing is inseparable from part-based processing as the emergent features within a face arise from the interrelations between isolated features. It also suggests that configural processing is more complex than part-based processing.

**Figure 2:**
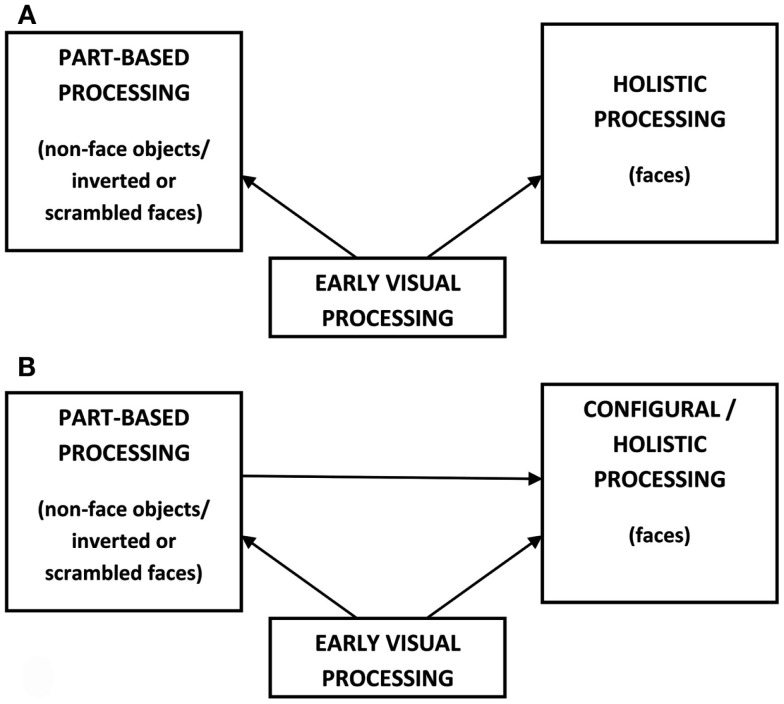
**Two different models of holistic processing**. **(A)** A face perception model adapted from Farah (1996). Object and face perception are independent of one another. Faces are perceived as undifferentiated wholes while objects are processed on the basis of their individual parts. **(B)** A *holistic/part-based model* of face perception. Holistic/configural and part-based processing work in parallel, making separate contributions to face perception that can be assessed independently of one another.

A third important term in the face perception literature is *holistic processing*. In its most pure sense, the term *holistic* implies the processing of an object as a series of templates, each of which cannot be broken down into parts or the interrelations between them. Face processing has been conceptualized this way (Tanaka and Farah, 1993; Farah, 1996). Supporters of this view argue that the perceptual processes used for object and face perception are dichotomous; faces are perceived as undifferentiated wholes while objects are processed on the basis of their individual parts (see Figure 2A). A major problem with adopting this purely holistic model of face perception is that in order to account for the many changes that can occur within a single face (e.g., viewpoint, expression, hairstyle) a multitude of templates would be needed of that individual’s face, which would require a very high memory load and may have implications for other cognitive and perceptual processes (Hoffman and Richards, 1984). There is also evidence to suggest that part-based processing does contribute to face recognition and that this can be assessed independently from some kind of configural/relational processing (as previously defined; e.g., McKone, 2004; Goffaux and Rossion, 2006). Overall then, a model of holistic processing with no decomposition into parts does not seem well supported.

More commonly in the face perception literature, the term *holistic* implies a specialized form of processing that involves the integration of all the information in a face, but does not preclude part-based processing (e.g., Rossion, 2008; McKone and Yovel, 2009). This will be referred to here as a *holistic/part-based model* (see Figure 2B) in which part-based and holistic processing act in parallel and both make important contributions during face perception. Advocates of this model believe that the holistic component of face processing can be isolated from the part-based component. Support for this has been found in studies where test stimuli are displayed in a subjects’ peripheral vision (e.g., McKone, 2004) or filtered to only include low spatial frequencies (e.g., Goffaux and Rossion, 2006) in conjunction with commonly used measures of holistic processing described below. Such techniques lead to feature details and boundaries becoming degraded, resulting in a greater reliance on the use of holistic information in discriminating between faces (Sergent, 1985). Interestingly, larger effects are found on measures of holistic processing when these degraded stimuli are used, lending support to the idea that holistic processing does not necessarily require detailed information about the features of a face (McKone, 2004; Goffaux and Rossion, 2006).

In the *holistic/part-based model*, holistic and configural processing are sometimes equated to mean the same thing (e.g., McKone and Yovel, 2009), implying that *all* of the configural information in a face must be processed/integrated at the same time. We favor a *holistic/part-based model* as it emphasizes that holistic and part-based processing are both separable and parallel processes that are equally important to face perception. In line with other advocates of this model the terms configural and holistic will be taken to mean the same thing here but only when configural processing involves the integration of all (as opposed to just some) of the spacing information between features.

However even within the *holistic/part-based model* there is a lack of consensus as to what constitutes the holistic representation of a face. Some versions view holistic processing as only including spacing differences between the features themselves, without including information about feature shape (which is instead processed in a part-based manner; Rossion, 2008). However recent reviews argue that there is a large body of evidence to suggest that feature shape information, instead of being coded separately, is also included in this representation (McKone and Yovel, 2009; Riesenhuber and Wolff, 2009; Yovel, 2009). Of course this has implications for the way in which the holistic representation of a face should be conceptualized in the face perception literature. In a conceptual model that is not inclusive of feature shape, feature center-points would be the only reliable markers for calculating configural information (McKone and Yovel, 2009). However these findings suggest that, feature spacing differences are calculated from key points surrounding the features themselves. These points are referred to as landmark (e.g., Rajapakse and Guo, 2001) or fiducial points (e.g., Tong et al., 2007). Alternatively it may be that the metric components of the holistic representation of a face are encoded more implicitly and do not rely on featural boundaries (McKone and Yovel, 2009).

Familiar (e.g., Young et al., 1987) and unfamiliar (e.g., Tanaka and Farah, 1993) faces have been used extensively with different measures of holistic processing. The next section contains a review of these different measures and how they have been used to explore holistic processing for faces.

## An Overview of Indirect and Direct Measures of Holistic Processing

Although the exact nature of holistic processing is still under debate, it is generally agreed that there exist a variety of paradigms that can be used to both measure holistic processing and manipulate the extent to which it is used (see McKone, 2010 or Tanaka and Gordon, 2011 for review). These measures fall into three main categories: indirect measures such as the disproportionate inversion effect; commonly used direct measures such as the composite task and part-whole task and; alternate measures of holistic processing that are not commonly used. These measures have been primarily used in conjunction with tasks that involve participants making identity related judgments about faces but there is also evidence to suggest holistic processing may be necessary for making other important face related judgments (e.g., expression and attractiveness) as well as identity judgments about bodies.

### The disproportionate inversion effect: An indirect measure of holistic processing

When faces and objects are turned upside down they become harder to recognize. This *inversion effect* is significantly larger for faces than it is for most other objects (Yin, 1969). However because this effect is disproportionate (i.e., larger for faces, but still occurs for objects), it cannot tell us whether there are qualitative differences between face and object processing (Valentine, 1988, 1991; McKone, 2010). For this reason the disproportionate inversion effect cannot be considered to directly measure holistic processing. One study has also shown indirect evidence of holistic processing in making attractiveness judgments, by showing the negative effect that inverting a face has on making reliable judgments about this type of attribution (Santos and Young, 2008). Face-sized inversion effects have also been shown for human bodies, at least with heads (Reed et al., 2003; Minnebusch et al., 2009; Yovel et al., 2010; Robbins and Coltheart, 2012a).

### The composite task and part-whole effect: Common direct measures of holistic processing

The following two paradigms assess and manipulate holistic processing directly and can be considered central to the face perception literature as they have also been used to show qualitative differences between upright and inverted faces and between faces and other objects (McKone, 2010).

**Figure 3:**
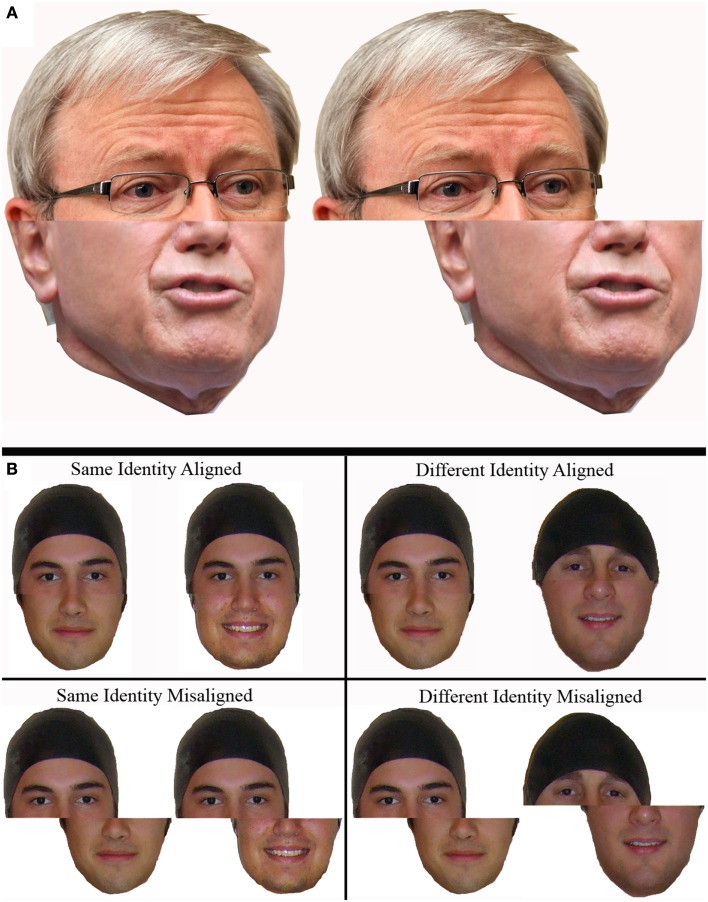
**The original versions of the composite task: (A) the naming version of the task used for familiar faces**. The top (or bottom) half of the composite is harder to identify when the halves are aligned (pictured left) as opposed to misaligned (pictured right). In this example the top half belongs to Kevin Rudd and the bottom half belongs to John Howard (both former Prime Ministers of Australia). **(B)** The same/different version using unfamiliar faces (where the top half is the half-to-match).

The *composite task* (Young et al., 1987; Hole, 1994; Le Grand et al., 2004), involves the use of stimuli created by joining together complementary halves of two different faces (usually the top and bottom halves). Two variants of the composite task are typically used depending on whether the faces are familiar or unfamiliar to the participant. In the familiar face version of the task, participants are asked to identify one half of a single composite face while ignoring the other (Young et al., 1987; see Figure 3A). Aligned face halves create the illusion of a new identity, making it harder to attend to one half of the face while ignoring the other. However this effect disappears when face halves are misaligned (vertically off-set). The unfamiliar face version of the composite task usually involves participants identifying whether the matching half of two composite faces (e.g., two top halves) belong to the same or two different individuals (e.g., Hole, 1994; Le Grand et al., 2004; see Figure 3B). There is some dispute over how holistic processing in this same/different version of the composite task should be measured. The original version uses differences in accuracy and/or reaction time between aligned and misaligned “same” trials to measure a composite effect (e.g., Le Grand et al., 2004; Robbins and McKone, 2007; Rossion and Boremanse, 2008). In this version the half-to-ignore is always from different people, making the predictions for “same” trials clear but the predictions for “different” trials unclear, which is why they are excluded. Specifically if two same facial halves are aligned with facial halves of two different people they will be perceived as looking less similar compared to when these composites are misaligned and integration does not occur. The extent to which the halves-to-match on different trials appear to be more or less similar is dependent on how alike the halves-to-be-ignored are. If different halves-to-ignore are very dissimilar, then when aligned with different halves-to-match they may actually look even *more different* than when misaligned. However if halves-to-ignore are very similar, then when aligned with different halves-to-match they may actually look *more similar* than when misaligned. As this likeness is almost impossible to control for, the data obtained from different trials is not used to measure for a composite effect when this approach is taken (McKone and Robbins, 2007).

An alternate version of the composite task uses both same and different trials to measure a composite effect (e.g., Gauthier et al., 1998). According to this method the extent to which an observer perceives two facial halves to be the same or different depends on whether the half-to-be-ignored requires the same decision (congruent) or a different decision (incongruent), resulting in four different ways in which composite stimuli can be paired when using this paradigm (half-to-match and half-to-ignore both same or both different, half-to-match same but half-to-ignore different, or half-to-match different but half-to-ignore same; Gauthier and Bukach, 2007). The measure of holistic processing is then usually taken not as the difference between aligned and misaligned trials, but as the difference between congruent and incongruent trials. The advocates of this version have argued that, for unfamiliar faces, the original composite effect is mainly decisional not perceptual (Richler et al., 2008). However, this alternate measure leads to bizarre results in which *misaligned* novel objects are apparently “holistically” processed (e.g., Wong et al., 2009). It also does not address the problem of predictions for different trials, meaning that effects may be diluted (or strengthened) in unpredictable ways.

On the standard measure of the composite task it has been shown that children as young as 3 months holistically process faces (Turati et al., 2010). In adults there is also a composite effect for human bodies (Robbins and Coltheart, 2012b) but there is no composite effect for car-fronts, novel objects, or dogs (the latter two even in experts; Cassia et al., 2009; Gauthier et al., 1998; Gauthier and Tarr, 2002; Robbins and McKone, 2007). Interestingly, for bodies the composite effect is larger for left-right integration, a form that has generally not been tested, compared to the more usual top-bottom integration (Robbins and Coltheart, 2012b). Robbins and Coltheart argue that this may indicate an important role for holistic processing in non-identity judgments such as attractiveness and communication, because integration for these judgments may be more important between left and right halves. For attractiveness (Abbas and Duchaine, 2008) and emotional expression (arguably a form of communication; Calder et al., 2000; White, 2000; Tanaka et al., 2012) only top-bottom integration has been tested but does suggest holistic processing. There is also evidence to suggest that holistic processing is used when making judgments about the gender (Baudouin and Humphreys, 2006), age (Hole and George, 2011), race (Michel et al., 2006), and perceived trustworthiness (Todorov et al., 2010) of a face.

Another commonly used measure of holistic processing is the part-whole task (Tanaka and Farah, 1993). In this paradigm participants are required to become familiar with a face (e.g., Bill) and then asked to either identify which of two faces, that differ only by one feature, shows the learned person (e.g., Bill versus Bill with Jim’s lips) or which of two facial features belongs to that particular person (e.g., Bill’s mouth versus Jim’s mouth). Participants are better at identifying features in the context of a whole face than in isolation, but not when faces are inverted or scrambled (Tanaka and Farah, 1993). This is because holistic integration creates the illusion that the new feature within the old face is a new face, making it easier to tell the two faces apart. However when the features are presented in isolation, or within an inverted or scrambled face, they must be discriminated in a part-based fashion, which is harder. A matching version of the task produces similar results (Davidoff and Donnelly, 1990; Donnelly and Davidoff, 1999).

A variation of the part-whole task involves also altering the spatial configuration between features (Tanaka and Sengco, 1997). By altering the spacing between the eyes, one’s ability to accurately recognize changes in the other features of the face diminishes. This is thought to occur because altering one source of information within the holistic representation of a face (i.e., the spacing between features) detrimentally affects the perception of other parts (including the individual features; Tanaka and Sengco, 1997; Tanaka and Gordon, 2011). Thus this task provides evidence of the importance of configural information within the holistic representation of a face.

Children show evidence of holistic processing on the spacing version of the part-whole task at age 4 (Pellicano et al., 2006). Both children aged 8–10 and adults also show similar sized part-whole effects for bodies as for faces (Seitz, 2002). Smaller or no part-whole effects are found for houses, chairs, novel objects, biological cells, and dog-faces (Davidoff and Donnelly, 1990; Tanaka and Farah, 1993; Tanaka et al., 1996, cited in Tanaka and Gauthier, 1997; Gauthier et al., 1998; Donnelly and Davidoff, 1999).

Overall, standard measures of holistic processing show effects for faces and perhaps human bodies but much smaller or no effect for other objects. They also show that holistic processing may develop reasonably early.

### Less commonly used measures of holistic processing

Other measures of holistic processing have either targeted the effects of masking or isolating specific regions of the face while making different types of discriminative judgments, or have explored the effect of inversion on discriminating between or detecting faces that have undergone different manipulations.

Tasks that involve masking, isolating, or drawing attention toward specific local regions of the face (e.g., nose region, eyebrow region) can provide a useful indication as to how much participants rely on the feature(s) in these regions to make different types of face-specific judgments (e.g., Gosselin and Schyns, 2001; Robbins and McKone, 2003; Sekuler et al., 2004; Santos and Young, 2011). The same process can also be applied to broader facial regions that contain more configural information such as the internal (center area of the face where the most salient features are located) and external (outer area of the face including the chin, forehead, hairline, and ears) regions of the face (Santos and Young, 2011). If accuracy is higher for whole face trials than isolated region trials then this is suggestive of holistic processing (Santos and Young, 2011). As noted earlier it is also possible to directly manipulate holistic versus feature processing by techniques such as blurring or spatial filtering (to remove feature information; McKone, 2004; Goffaux and Rossion, 2006) or scrambling of features (to remove holistic configuration; Tanaka and Farah, 1993).

Common measures of holistic processing produce effects for upright faces (e.g., Young et al., 1987; Tanaka and Farah, 1993), but effects are greatly reduced or absent for inverted faces. Because of this, information that becomes harder to detect when a face is displayed upside down (whether it is a single feature or more relational/configural in nature) has been argued to form part of the holistic representation of that face (e.g., McKone and Yovel, 2009). Detecting changes to either the spacing (e.g., Goffaux and Rossion, 2006) or feature shape information (see McKone and Yovel, 2009, for review), within a face is significantly harder to do when faces are inverted. The difference between upright and inverted faces with manipulations to spacing and/or features has then also been used to measure holistic processing in other-race faces, for example. Children show the ability to detect spacing changes to faces from 5 months of age (e.g., Bhatt et al., 2005), but larger changes are usually needed than for adults (see review in Mondloch and Thomson, 2008).

Other paradigms such as the superimposed faces task (Martini et al., 2006) and the Mooney face task (McKone, 2004) have also been used as measures of holistic processing, but because the nature of these tasks involve detecting faces (or salience differences between faces) instead of making different types of discriminations between these faces, they could be argued to be more representative of a face detection stage, rather than a face discrimination stage of holistic processing (see Tsao and Livingstone, 2008 for review).

Overall, measures of holistic processing suggest faces and bodies are processed differently to other objects and there are a variety of ways of tapping into this difference. Further, holistic processing may be used for not only identity but also other judgments such as attractiveness, expression, age, gender, and social attributions such as trustworthiness. Robbins and Coltheart (2012b) particularly suggest that examining left-right integration as well as top-bottom integration could lead to important increases in understanding of the nature of holistic processing. They predict, for example that for attractiveness holistic processing may be stronger for left-right integration because of the importance of symmetry to judgments of attractiveness. Next we consider another aspect of faces that may greatly increase our understanding of holistic processing.

## What Can Moving Faces Tell Us About Holistic Processing?

When we see faces and bodies in real life, they are usually engaged in some type of motion. However the vast majority of studies using the above measures of holistic processing have only used static faces as stimuli. Although the role and effectiveness of both structural and dynamic information in recognizing faces has been explored extensively in the literature, very few studies have applied measures of configural and holistic processing to moving facial stimuli. In relation to making identity judgments from moving faces, there are only two inversion effect studies focusing on famous faces engaging in conversation (Knight and Johnston, 1997; Lander et al., 1999) and only one study that has applied the composite task to unfamiliar faces engaging in rigid motion (Xiao et al., 2012). In addition to this there has been one study that has explored inversion effects for subtle facial expression processing for static and moving faces (Ambadar et al., 2005). Using moving, as opposed to static, facial stimuli on measures of holistic processing may help to clarify conceptual issues regarding what is included in the holistic representation of a face; improve the ease with which the components of a face are processed as a whole and; enhance face perception by providing additional information about a face.

Facial motion can be rigid or non-rigid and in most cases human interactions involve a combination of both types of movement (O’Toole et al., 2002; Roark et al., 2003). Rigid motion includes turning, nodding, and shaking of the head (Roark et al., 2003). Rigid motion is also apparent when moving past a still person/object. Non-rigid motion refers to the elastic change that can occur within the features of a face (e.g., moving the lips in conversation; O’Toole et al., 2002; Roark et al., 2003).

Within the unfamiliar facial recognition literature there are conflicting results concerning how much additional information movement provides for face recognition, over the use of still images. Some studies have found no such motion advantage for any type of facial movement (e.g., Christie and Bruce, 1998), while others have found a beneficial role for rigid motion (e.g., Pike et al., 1997), non-rigid motion (e.g., Thornton and Kourtzi, 2002), or both types of motion (e.g., Lander and Bruce, 2003). Differences in these results may, in part, be explained by the different types of tasks used. These included old/new recognition memory tasks (e.g., Lander and Bruce, 2003), sequential matching tasks (Thornton and Kourtzi, 2002), and a “delayed visual search paradigm” (Pilz et al., 2006, 2009).

Only one study has applied a direct measure of holistic processing to moving faces, and focused only on rigidly moving heads during the learning phase of the experiment (i.e., the test phase included only static faces; Xiao et al., 2012). This study found no composite effect for temporally coherent rigid head movement (heads rotating in view) but found composite effects for both temporally incoherent (random frames of different views) and temporally separate (visual noise separating frames) photo sequences. From these findings Xiao and colleagues concluded that people were more likely to use part-based than holistic processing in recognition tasks involving rigid head movement. A different study focusing on holistic processing and changes in facial viewpoint in static faces arrived at a similar conclusion, finding an increased reliance on part-based processing for some facial viewpoints but not others using inversion and feature scrambling techniques and displaying these in the periphery (McKone, 2008). However, McKone (2008) also found no difference in holistic processing at three different viewpoints [frontal (0°), three-quarter (45°), or profile (90°)] using the composite task. Together these results, although not conclusive, suggest that rigidly moving faces may produce weaker effects on measures of holistic processing than static faces. This may result from a greater dependence on featural (part-based) information in identifying faces due to apparent changes within its second-order configuration.

Indirect evidence of holistic processing for moving face stimuli has also been found using the inversion task (Knight and Johnston, 1997; Lander et al., 1999). These studies found similar sized inversion effects when participants either identified celebrities from still frames or from dynamic video sequences, suggesting that holistic processing may operate in similar ways for static and dynamic faces. Inversion effects have also been found for moving faces in judging subtle facial expression (Ambadar et al., 2005). However the size of the inversion effect was no different to that found when the same expression judgments were made from static faces. Overall these studies do not suggest increased holistic processing for moving faces but more research is needed in the area.

Using rigid and non-rigid moving faces in conjunction with common measures of holistic processing may help to clarify conceptual issues regarding what is included in the holistic representation of a face (c.f. McKone and Yovel, 2009). There would be challenges in using standard measures with moving faces (e.g., getting facial halves from two different people to remain aligned as well as move in unison with one another). However existing paradigms would serve as a useful starting point in the exploration of holistic processing for moving faces and the challenges that arise from this task may give way to alternate measures.

**Figure 4:**
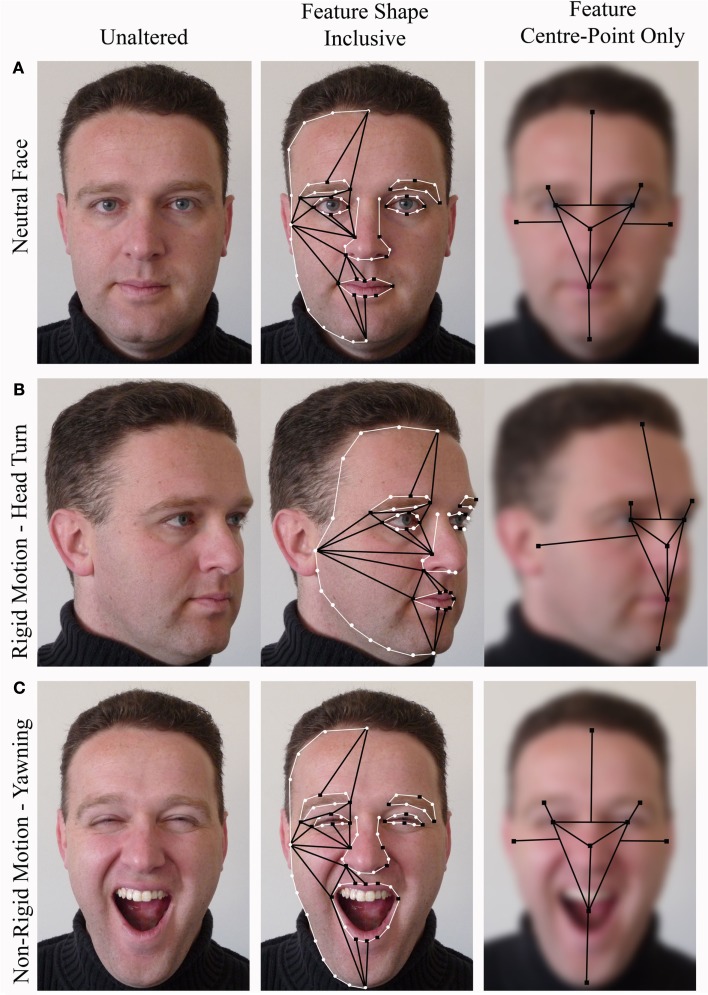
**Examples of unaltered, feature shape inclusive and feature center point only conceptual models [based on models proposed by McKone and Yovel (2009)] for (A) a neutral face, (B) a turning face, and (C) a yawning face**. Black squares represent key points from which configural information is calculated, black lines represent configural information, white circles represent other contour points, and white lines follow the shapes of the features. Center-point only images have been blurred to emphasize that feature shape does not factor into the holistic representation in this model.

Regardless of whether the holistic representation of a face is thought to be inclusive of feature shape (McKone and Yovel, 2009; Riesenhuber and Wolff, 2009; Yovel, 2009) or not (Rossion, 2008), any motion that includes viewpoint change (e.g., turning the head) should lead to apparent changes in the configuration of a face (Compare Figures 4A,B). Based on this one might predict that a turning or nodding face, as opposed to a static one, may lead to some sort of quantitative change on measures of holistic and configural processing. However predictions about the effect of feature (as opposed to whole head) movement on holistic processing would depend on whether feature shape was included in the holistic representation of a face (c.f. McKone and Yovel, 2009). To provide an example of this, imagine a situation in which an unfamiliar person yawns while you are processing their face (Compare Figures 4A,C). You might expect to see their mouth widening and their eyes narrowing (among other changes) as they begin to yawn. If the holistic representation of a face was inclusive of feature shape and was measured from feature boundaries, you would expect several changes to occur in the second-order configuration of that face. For instance the lower mouth boundary (the bottom lip) would move further away from the nose and eye boundaries. However if feature shape was not included in the holistic representation of a face (c.f. Rossion, 2008), and relational information was instead measured from the center-points of shapeless features, then little change would occur in the second-order configuration of that face. The center-point of the mouth would change little in relation to other feature center-points regardless of how far the mouth was opened. If the holistic representation of a face is inclusive of changes in feature shape then one might expect to see some sort of quantitative difference between faces with moving features and still faces on measures of holistic and configural processing. If the holistic representation of a face is not inclusive of changes in feature shape then one might expect to see little or no quantitative difference between these two types of stimuli.

Using moving as opposed to static faces on these measures may also allow the face to be perceived more easily and effectively as a whole due to the applicability of additional Gestalt grouping principals specific to moving stimuli. When faces are still, their features possess a range of properties that allow them to be grouped together effectively as a whole. These include the “proximity” of the features within the face, the “common region” they share (i.e., the face itself) and the approximate bilateral “symmetry” generated by the shape, positioning, and spacing of facial features when they are seen from a frontal viewpoint. When faces engage in motion two additional Gestalt grouping principles can be applied to their features. The first is the “common fate” shared by the facial features when they move in the same direction and at the same pace during rigid motion and the second is the “synchrony” generated by the movement of different facial features at the same time (but in different ways) during non-rigid motion (see Wagemans et al., 2012a, for a comprehensive overview of Gestalt grouping principles).

In line with the “representation enhancement hypothesis” (O’Toole et al., 2002; Roark et al., 2003), it is also possible that the use of moving faces on these measures may enhance facial perception and recognition in a variety of other ways. Rigid motion may assist in holistic processing because it not only provides additional exemplars of that face through viewpoint change but may also allow for a 3D representation of that face to be developed, allowing it to be identified from previously unseen viewpoints (Christie and Bruce, 1998). Rigid and non-rigid motion may also assist in facial processing by revealing information about how much the internal features of a face and their spatial configuration can vary during different types of movement (Christie and Bruce, 1998). For example when a face is engaged in conversation (with minimal to no head movement) the distance between the upper and lower boundaries of the mouth would vary considerably whereas the distance between inner corners of the eyes would remain invariant. However during head rotation along the yaw axis the distance between the upper and lower mouth boundaries would remain relatively invariant while the apparent distance between the eyes would change.

Studying faces in motion, therefore will allow us to determine the information most relevant to the perception of a face as a whole. Exploring the joint contribution of facial structure and motion to holistic processing will also allow facial processing to be explored in a more naturalistic context. This will potentially lead to a clearer understanding about how holistic processing operates in the real world. If holistic processing was found to operate in a similar way for both still and moving faces then such experiments would serve to strengthen the validity of existing research in the field of facial processing. However if holistic processing for moving faces was somehow different from what has previously been found for static faces, then the ecological validity of existing findings with static faces would be put into question and theories of face recognition might need to be rethought.

## Conclusion

This review article has traced the routes of holistic processing from Gestalt theory, distinguishing between commonly referenced models of face perception and how they differ from a purely part-based model of perception. Despite the wide array of research in the face perception literature, further clarification is needed in the field. We have reviewed both common and lesser known measures of holistic processing. These measures support a holistic/part-based model of face perception whereby holistic and part-based processing make parallel and separable contributions to face perception. Both the individual features and the holistic representation of a face appear relevant to face perception, however it is still uncertain what is included in the holistic representation. Establishing whether feature shape plays a role in the holistic representation of a face will help determine whether configural information is measured from the center-points of shapeless blobs or from key points surrounding the featural boundaries. Using moving facial stimuli with existing measures of holistic processing should provide a useful means of answering this question. The use of moving faces in this field should also allow facial processing to be explored in a way more similar to how it occurs in the real world where faces relay socially relevant information through movement.

## Conflict of Interest Statement

The authors declare that the research was conducted in the absence of any commercial or financial relationships that could be construed as a potential conflict of interest.

## References

[B1] Abbas Z. Duchaine B. (2008). The role of holistic processing in judgments of facial attractiveness. Perception 37, 1187–1196 10.1068/p5984 18853555

[B2] Ambadar Z. Schooler J. W. Cohn J. (2005). Deciphering the enigmatic face: the importance of facial dynamics in interpreting subtle facial expressions. Psychol. Sci. 16, 403–410 10.1111/j.0956-7976.2005.01548.x 15869701

[B3] Bartlett J. Searcy J. H. Abdi H. (2003). “What are the routes to face recognition?” in Perception of Faces, Objects, and Scenes: Analytic and Holistic Processes, eds Peterson M. Rhodes G. (Oxford: Oxford University Press), 21–52

[B4] Baudouin J. Y. Humphreys G. W. (2006). Configural information in gender categorisation. Perception 35, 531–540 10.1068/p3403 16700294

[B5] Bhatt R. S. Bertin E. Hayden A. Reed A. (2005). Face processing in infancy: developmental changes in the use of different kinds of relational information. Child Dev. 76, 169–181 10.1111/j.1467-8624.2005.00837.x 15693765

[B6] Biederman I. (1987). Recognition-by-components: a theory of human image understanding. Psychol. Rev. 94, 115–147 10.1037/0033-295X.94.2.115 3575582

[B7] Calder A. J. Young A. W. Keane J. Dean M. (2000). Configural information in facial expression perception. J. Exp. Psychol. Hum. Percept. Perform. 26, 527–551 10.1037/0096-1523.26.2.527 10811161

[B8] Cassia V. M. Picozzi M. Kuefner D. Bricolo E. Turati C. (2009). Holistic processing for faces and cars in preschool-aged children and adults: evidence from the composite effect. Dev. Sci. 12, 236–248 10.1111/j.1467-7687.2008.00765.x 19143797

[B9] Christie F. Bruce V. (1998). The role of dynamic information in the recognition of unfamiliar faces. Mem. Cognit. 26, 780–790 10.3758/BF03211397 9701969

[B10] Davidoff J. Donnelly N. (1990). Object superiority: a comparison of complete and part probes. Acta Psychol. (Amst.) 73, 225–243 10.1016/0001-6918(90)90024-A 2353588

[B11] Diamond R. Carey S. (1986). Why faces are and are not special: an effect of expertise. J. Exp. Psychol. Gen. 115, 107–117 10.1037/0096-3445.115.2.107 2940312

[B12] Donnelly N. Davidoff J. (1999). The mental representations of faces and houses: issues concerning parts and wholes. Vis. Cogn. 6, 319–343 10.1080/135062899395000

[B13] Duchaine B. C. Yovel G. Butterworth E. J. Nakayama K. (2006). Prosopagnosia as an impairment to face-specific mechanisms: elimination of the alternative hypotheses in a developmental case. Cogn. Neuropsychol. 23, 714–747 10.1080/02643290600631515 21049351

[B14] Farah M. J. (1996). Is face recognition ‘special’? Evidence from neuropsychology. Behav. Brain Res. 76, 181–189 10.1016/0166-4328(95)00198-0 8734052

[B15] Gauthier I. Bukach C. (2007). Should we reject the expertise hypothesis? Cognition 103, 322–330 10.1016/j.cognition.2006.03.002 16780825

[B16] Gauthier I. Tarr M. J. (2002). Unravelling mechanisms for expert object recognition: bridging brain activity and behaviour. J. Exp. Psychol. Hum. Percept. Perform. 28, 431–446 10.1037/0096-1523.28.2.431 11999864

[B17] Gauthier I. Williams P. Tarr M. J. Tanaka J. (1998). Training ‘greeble’ experts: a framework for studying expert object recognition processes. Vision Res. 38, 2401–2428 10.1016/S0042-6989(97)00442-2 9798007

[B18] Goffaux V. Rossion B. (2006). Faces are “spatial” – holistic face perception is supported by low spatial frequencies. J. Exp. Psychol. Hum. Percept. Perform. 32, 1023–1039 10.1037/0096-1523.32.4.1023 16846295

[B19] Gosselin F. Schyns P. G. (2001). Bubbles: a technique to reveal the use of information in recognition tasks. Vision Res. 41, 2261–2271 10.1016/S0042-6989(01)00097-9 11448718

[B20] Hoffman D. D. Richards W. A. (1984). Parts of recognition. Cognition 18, 65–69 10.1016/0010-0277(84)90022-2 6543164

[B21] Hole G. George P. (2011). Evidence for holistic processing of facial age. Vis. Cogn. 19, 585–615 10.1080/13506285.2011.562076

[B22] Hole G. J. (1994). Configurational factors in the perception of unfamiliar faces. Perception 23, 65–74 10.1068/p230065 7936977

[B23] Johnson M. H. Dziurawiec S. Ellis H. Morton J. (1991). Newborns’ preferential tracking of face-like stimuli and its subsequent decline. Cognition 40, 1–19 10.1016/0010-0277(91)90045-6 1786670

[B24] Kanwisher N. Yovel G. (2006). The fusiform face area: a cortical region specialized for the perception of faces. Philos. Trans. R. Soc. Lond. B Biol. Sci. 361, 2109–2128 10.1098/rstb.2006.1934 17118927 PMC1857737

[B25] Knight B. Johnston A. (1997). The role of movement in face recognition. Vis. Cogn. 4, 265–273 10.1080/713756764

[B26] Lander K. Bruce V. (2003). The role of motion in learning new faces. Vis. Cogn. 10, 897–912 10.1080/13506280344000149

[B27] Lander K. Christie F. Bruce V. (1999). The role of movement in the recognition of famous faces. Mem. Cognit. 27, 974–985 10.3758/BF03201228 10586574

[B28] Latimer C. Stevens C. (1997). Some remarks on wholes, parts and their perception. Psycoloquy 8, 1–14

[B29] Le Grand R. Mondloch C. J. Maurer D. Brent H. P. (2004). Impairment in holistic face processing following early visual deprivation. Psychol. Sci. 15, 762–768 10.1111/j.0956-7976.2004.00753.x 15482448

[B30] Leder H. Bruce V. (2000). When inverted faces are recognized: the role of configural information in face recognition. Q. J. Exp. Psychol. 53A, 513–536 10.1080/713755889 10881616

[B31] Martini P. McKone E. Nakayama K. (2006). Orientation tuning of human face processing estimated by contrast matching in transparency displays. Vision Res. 46, 2102–2109 10.1016/j.visres.2005.11.014 16384592

[B32] Maurer D. Le Grand R. Mondloch C. J. (2002). The many faces of configural processing. Trends Cogn. Sci. (Regul. Ed.) 6, 255–260 10.1016/S1364-6613(02)01903-4 12039607

[B33] McKone E. (2004). Isolating the special component of face recognition: peripheral identification and a mooney face. J. Exp. Psychol. Learn. Mem. Cogn. 30, 181–197 10.1037/0278-7393.30.1.181 14736306

[B34] McKone E. (2008). Configural processing and face viewpoint. J. Exp. Psychol. Hum. Percept. Perform. 34, 310–327 10.1037/0096-1523.34.2.310 18377173

[B35] McKone E. (2010). “Face and object recognition: how do they differ?” in Tutorials in Visual Cognition, ed. Coltheart V. (New York, NY: Psychology Press), 261–304

[B36] McKone E. Crooks K. Jeffery L. Dilks D. D. (2012). A critical review of the development of face recognition: experience is less important than previously believed. Cogn. Neuropsychol. 29, 174–212 10.1080/02643294.2012.660138 22360676

[B37] McKone E. Robbins R. (2007). The evidence rejects the expertise hypothesis: reply to Gauthier & Bukach. Cognition 103, 331–336 10.1016/j.cognition.2006.05.014 16842769

[B38] McKone E. Robbins R. (2011). “Are faces special?” in The Oxford Handbook of Face Perception, eds Calder A. J. Rhodes G. Johnson M. H. Haxby J. V. (New York, NY: Oxford University Press), 149–176

[B39] McKone E. Yovel G. (2009). Why does picture-plane inversion sometimes dissociate perception of features and spacing in faces, and sometimes not? Toward a new theory of holistic processing. Psychon. Bull. Rev. 16, 778–797 10.3758/PBR.16.5.778 19815781

[B40] Michel C. Rossion B. Han J. Chung C. Caldara R. (2006). Holistic processing is finely tuned for faces of one’s own race. Psychol. Sci. 17, 608–615 10.1111/j.1467-9280.2006.01752.x 16866747

[B41] Minnebusch D. A. Suchan B. Daum I. (2009). Losing your Head: behavioral and electrophysiological effects of body inversion. J. Cogn. Neurosci. 21, 865–874 10.1162/jocn.2009.21074 18702581

[B42] Mondloch C. J. Thomson K. (2008). Limitations in 4-year-old children’s sensitivity to the spacing among facial features. Child Dev. 79, 1514–1524 10.1111/j.1467-8624.2008.01202.x 18826539

[B43] O’Toole A. J. Roark D. A. Abdi H. (2002). Recognizing moving faces: a psychological and neural synthesis. Trends Cogn. Sci. (Regul. Ed.) 6, 261–266 10.1016/S1364-6613(02)01908-3 12039608

[B44] Pellicano E. Rhodes G. Peters M. (2006). Are preschooolers sensitive to configural information in faces? Dev. Sci. 9, 270–277 10.1111/j.1467-7687.2006.00489.x 16669797

[B45] Pike G. E. Kemp R. I. Towell N. A. Phillips K. C. (1997). Recognizing moving faces: the relative contribution of motion and perspective view information. Vis. Cogn. 4, 409–437 10.1080/713756769

[B46] Pilz K. S. Bülthoff H. H. Vuong Q. C. (2009). Learning influences the encoding of static and dynamic faces and their recognition across different spatial frequencies. Vis. Cogn. 17, 716–735 10.1080/13506280802340588

[B47] Pilz K. S. Thornton I. M. Bülthoff H. H. (2006). A search advantage for faces learned in motion. Exp. Brain Res. 171, 436–447 10.1007/s00221-005-0283-8 16331505

[B48] Rajapakse M. Guo Y. (2001). “Multiple landmark feature point mapping for robust face recognition,” in Proceedings of Audio and Video-Based Biometric Person Authentication, Third International Conference, AVBPA 2001, eds Bigün J. Smeraldi F. (Berlin: Springer), 96–101

[B49] Reed C. L. Stone V. E. Bozova S. Tanaka J. (2003). The body-inversion effect. Psychol. Sci. 14, 302–308 10.1111/1467-9280.14431 12807401

[B50] Rhodes G. (1988). Looking at faces: first-order and second-order features as determinants of facial appearance. Perception 17, 43–63 10.1068/p170043 3205669

[B51] Richler J. J. Gauthier I. Wenger M. J. Palmeri T. J. (2008). Holistic processing of faces: perceptual and decisional components. J. Exp. Psychol. Learn. Mem. Cogn. 34, 328–342 10.1037/0278-7393.34.2.328 18315409

[B52] Riesenhuber M. Wolff B. S. (2009). Task effects, performance levels, features, configurations, and holistic face processing: a reply to Rossion. Acta Psychol. (Amst.) 132, 286–292 10.1016/j.actpsy.2009.07.004 19665104 PMC2788156

[B53] Roark D. A. Barret S. E. Spence M. Abdi H. O’Toole A. J. (2003). Memory for moving faces: psychological and neural perspectives on the role of motion in face recognition. Behav. Cogn. Neurosci. Rev. 2, 15–46 10.1177/1534582303002001002 17715597

[B54] Robbins R. A. Coltheart M. (2012a). The effects of inversion and familiarity on face versus body cues to person recognition. J. Exp. Psychol. Hum. Percept. Perform. 38, 1098–1104 10.1037/a0028584 22642217

[B55] Robbins R. A. Coltheart M. (2012b). Left–right holistic integration of human bodies. Q. J. Exp. Psychol. 65, 1962–1974 10.1080/17470218.2012.674145 22524623

[B56] Robbins R. McKone E. (2003). Can holistic processing be learned for inverted faces? Cognition 88, 79–107 10.1016/S0010-0277(03)00020-9 12711154

[B57] Robbins R. McKone E. (2007). No face-like processing for objects-of-expertise in three behavioural tasks. Cognition 103, 34–79 10.1016/j.cognition.2006.02.008 16616910

[B58] Rossion B. (2008). Picture-plane inversion leads to qualitative changes of face perception. Acta Psychol. (Amst.) 128, 274–289 10.1016/j.actpsy.2008.02.003 18396260

[B59] Rossion B. Boremanse A. (2008). Nonlinear relationship between holistic processing of individual faces and picture-plane rotation: evidence from the face composite illusion. J. Vis. 8, 1–13 10.1167/8.8.1 18484842

[B60] Santos I. M. Young A. W. (2008). Effects of inversion and negation on social inferences from faces. Perception 37, 1061–1078 10.1068/p5278 18773729

[B61] Santos I. M. Young A. W. (2011). Inferring social attributes from different face regions: evidence for holistic processing. Q. J. Exp. Psychol. 64, 751–766 10.1080/17470218.2010.519779 21086218

[B62] Seitz K. (2002). Parts and wholes in person recognition: developmental trends. J. Exp. Child. Psychol. 82, 367–381 10.1016/S0022-0965(02)00106-6 12225760

[B63] Sekuler A. B. Gaspar C. M. Gold J. M. Bennett P. J. (2004). Inversion leads to quantitative, not qualitative changes in face processing. Curr. Biol. 14, 391–396 10.1016/j.cub.2004.02.028 15028214

[B64] Sergent J. (1985). Influence of task and input factors on hemispheric involvement in face processing. J. Exp. Psychol. Hum. Percept. Perform. 11, 846–861 10.1037/0096-1523.11.6.846 2934512

[B65] Tanaka J. W. Farah M. J. (1993). Parts and wholes in face recognition. Q. J. Exp. Psychol. A 46, 225–245 10.1080/14640749308401045 8316637

[B66] Tanaka J. W. Gauthier I. (1997). Expertise in object and face recognition. Psychol. Learn. Mot. 36, 83–125 10.1016/S0079-7421(08)60282-0

[B67] Tanaka J. W. Gordon I. (2011). “Features, configuration, and holistic face processing,” in The Oxford Handbook of Face Perception, eds Calder A. J. Rhodes G. Johnson M. H. Haxby J. V. (New York, NY: Oxford University Press), 149–176

[B68] Tanaka J. W. Kaiser M. D. Butler S. Le Grand R. (2012). Mixed emotions: holistic and analytic perception of facial expressions. Cogn. Emot. 26, 961–977 10.1080/02699931.2011.630933 22273429

[B69] Tanaka J. W. Sengco J. A. (1997). Features and their configuration in face recognition. Mem. Cognit. 25, 583–592 10.3758/BF03211301 9337578

[B70] Thornton I. M. Kourtzi Z. (2002). A matching advantage for dynamic human faces. Perception 31, 113–132 10.1068/p3072 11922118

[B71] Todorov A. Loehr V. Oosterhof N. N. (2010). The obligatory nature of holistic processing of faces in social judgments. Perception 39, 514–532 10.1068/p6501 20514999

[B72] Tong Y. Wang Y. Zhu Z. Ji Q. (2007). Robust facial feature tracking under varying face pose and facial expression. Pattern Recognit. 40, 3195–3208 10.1016/j.patcog.2007.02.021

[B73] Tsao D. Y. Livingstone M. S. (2008). Mechanisms of face perception. Annu. Rev. Neurosci. 31, 411–437 10.1146/annurev.neuro.30.051606.094238 18558862 PMC2629401

[B74] Turati C. Di Giorgio E. Bardi L. Simion F. (2010). Holistic face processing in newborns, 3-month-old infants, and adults: evidence from the composite face effect. Child Dev. 81, 1894–1905 10.1111/j.1467-8624.2010.01520.x 21077871

[B75] Valentine T. (1988). Upside-down faces: a review of the effect of inversion upon face recognition. Br. J. Psychol. 79, 471–491 10.1111/j.2044-8295.1988.tb02747.x 3061544

[B76] Valentine T. (1991). A unified account of the effects of distinctiveness, inversion and race in face recognition. Q. J. Exp. Psychol. A 43, 161–204 10.1080/14640749108400966 1866456

[B77] Wagemans J. Elder J. H. Kubovy M. Palmer S. E. Peterson M. A. Singh M. (2012a). A century of Gestalt psychology in visual perception: I. Perceptual grouping and figure-ground organization. Psychol. Bull. 138, 1172–1217 10.1037/a0029333 22845751 PMC3482144

[B78] Wagemans J. Feldman J. Gepshtein S. Kimchi R. Pomerantz J. R. van der Helm P. A. (2012b). A century of Gestalt psychology in visual perception: II. Conceptual and theoretical foundations. Psychol. Bull. 138, 1218–1252 10.1037/a0029333 22845750 PMC3728284

[B79] White M. (2000). Parts and wholes in expression recognition. Cogn. Emot. 14, 39–60 10.1080/026999300378987

[B80] Wong A. C. N. Palmeri T. J. Gauthier I. (2009). Conditions for facelike expertise with objects: becoming a ziggerin expert – but which type? Psychol. Sci. 20, 1108–1117 10.1111/j.1467-9280.2009.02430.x 19694980 PMC2919853

[B81] Xiao N. G. Quinn P. C. Ge L. Lee K. (2012). Rigid facial motion influences featural, but not holistic, face processing. Vision Res. 57, 26–34 10.1016/j.visres.2012.01.015 22342561 PMC3302942

[B82] Yin R. K. (1969). Looking at upside-down faces. J. Exp. Psychol. 81, 141–145 10.1037/h0027474

[B83] Young A. W. Hellawell D. Hay D. C. (1987). Configurational information in face perception. Perception 16, 747–759 10.1068/p160747 3454432

[B84] Yovel G. (2009). The shape of facial features and the spacing among them generate similar inversion effects: a reply to Rossion (2008). Acta Psychol. (Amst.) 132, 293–299 10.1016/j.actpsy.2009.07.009 19666168

[B85] Yovel G. Pelc T. Lubetzky I. (2010). It’s all in your head: why is the body inversion effect abolished for headless bodies? J. Exp. Psychol. Hum. Percept. Perform. 36, 759–767 10.1037/a0017451 20515202

